# Inner Ear Organoids: Strengths and Limitations

**DOI:** 10.1007/s10162-024-00929-2

**Published:** 2024-02-09

**Authors:** Giulia Pianigiani, Marta Roccio

**Affiliations:** 1 Institute for Maternal and Child Health – I.R.C.C.S. “Burlo Garofolo”, Trieste, Italy; 2 Inner Ear Stem Cell Lab, Department Otorhinolaryngology, Head and Neck Surgery, University Hospital Zurich and University of Zurich, USZ Campus WAGI18, Wagistrasse 18, 8952 Schlieren, Switzerland

**Keywords:** Human iPSC-derived organoids, Inner ear biology, Hearing loss

## Abstract

Inner ear organoids derived from differentiation of human pluripotent stem cells have recently gained momentum as tools to study inner ear development and developmental defects. An additional exciting aspect about this technology is represented by its translational potential, specifically, the use of organoids to validate therapeutics for hearing and balance restoration on human/patient-specific cells. This latter aspect will be briefly discussed here including opportunities and current limitations.

The difficult access to human inner ear tissue significantly hinders gaining molecular insights into organ biology and pathophysiology. This information is however critical for diagnostic and therapeutic purposes. Animal models enable to correlate and address causality between altered physiological responses and molecular/cellular defects, yet, in many cases, the transferability of the findings to humans remains unverified. Cellular models of the inner ear derived from tissue progenitors, stem cell differentiation, or direct reprograming, represent alternative tools to derive human/patient-specific cells, bypassing, for the latter approaches, the hurdles of primary tissue collection [1]. These ex vivo culture systems could be used for disease modeling and for in vitro validation of novel therapies. A number of recent reviews has been published discussing advantages and limitations of many of these approaches, including the history of their development and potential applications [2–6]. We invite the readers to take a look at this literature to gain a better overview of this booming field of research. In this opinion article, we cover exclusively human pluripotent stem cell-derived inner ear organoids. We address the most recent advancements of this technology and provide a candid review of their limitations.

## Pluripotent Stem Cell–Derived Inner Ear Organoids (IEOs)

The combination of a three-dimensional culture system and small molecules/morphogens**–**guided differentiation enables the differentiation of mouse and human pluripotent stem cells—including embryonic stem cells (ESCs) and induced pluripotent stem cells (iPSCs)—to bona fide inner ear cell types in the so-called inner ear organoids (IEOs) [7–10]. Sensory epithelia generated in IEOs display remarkable similarities to inner ear organs, in particular vestibular epithelia, with respect to hair cell shape, intercalation of hair cells and supporting cells, hair bundle development, neuronal innervation, and transcriptome. Careful electrophysiological characterization of the murine IEOs determined that hair cells have specific vestibular features [11]. More recently, using a modified guidance protocol, it has been demonstrated that it is possible to promote the differentiation of cochlear-like hair cells, with the bundle morphology, marker expression, and transcriptional identity of human fetal cochlear hair cells [12].

**Fig. 1:**
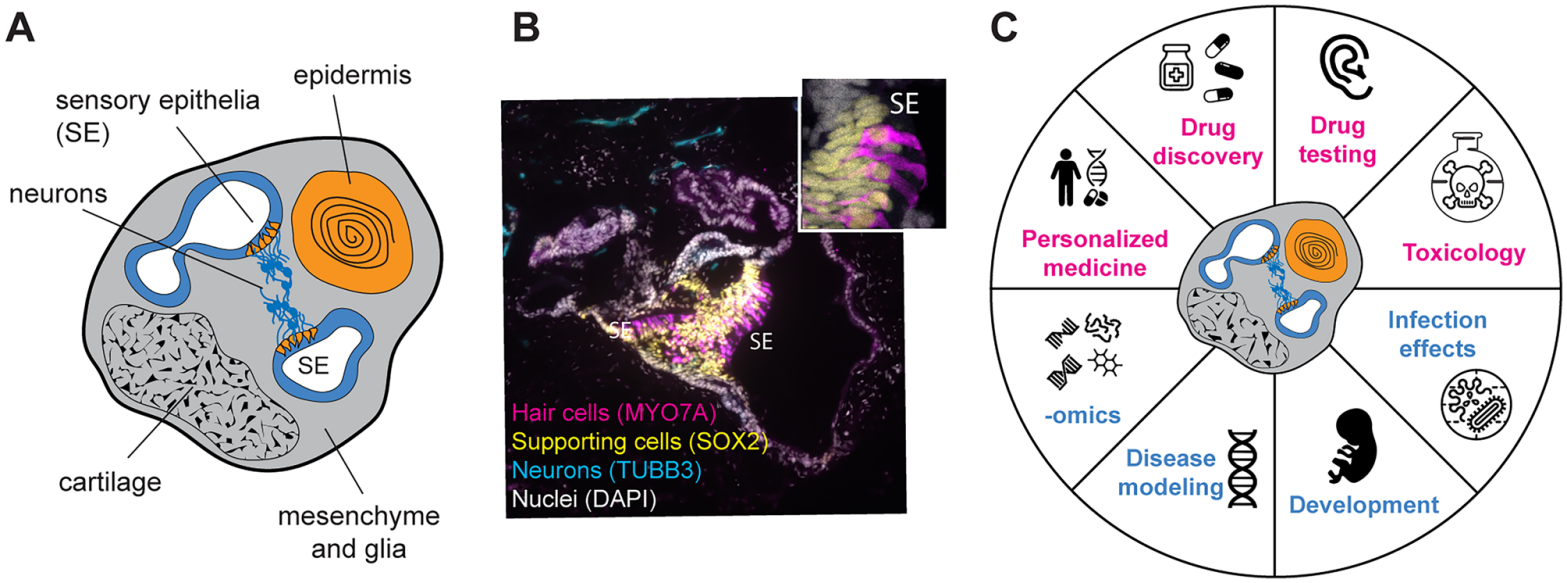
**A** IEOs contain different otic cell types: sensory epithelia (SE), non-sensory epithelia, neurons, glia, and mesenchyme, but also off-target tissues such as cartilage and epidermis. **B** Representative example of otic vesicle with sensory epithelium containing MYO7A + hair cells, SOX2 + supporting cells, and TUBB3 + neurons. **C** Application of IEOs technology: present (blue) and future (pink)

IEOs have several potential applications, many of which have been previously reviewed [2–4, 13] (Fig. 1). Here, we only highlight some points of consideration.

A recent set of papers has provided an in-depth characterization of human iPSC-derived IEOs and a first comparison with human inner ear embryonic and fetal samples [12, 14–18]. By comparing single-cell profiling of IEOs, human fetal inner ear and adult vestibular epithelia, as well as by histological comparison, these studies established that in vitro differentiation follows similar timing and dynamics as in vivo. Cells differentiated in IEOs for 50–70 days match marker expression and maturity of weeks 10–12 human development [14, 17]. Further differentiation in culture until days 150–200 brings the maturity of the culture closer to weeks 18–20 of fetal inner ear development, when functionality starts [12]. These studies represent an important step forward for the field; however, more complete human inner ear embryonic/fetal/adult atlases are needed as a reference, in order for these types of studies to give a full picture of the differentiating sensory epithelia and ganglia during human development [19, 20].

**Fig. 2:**
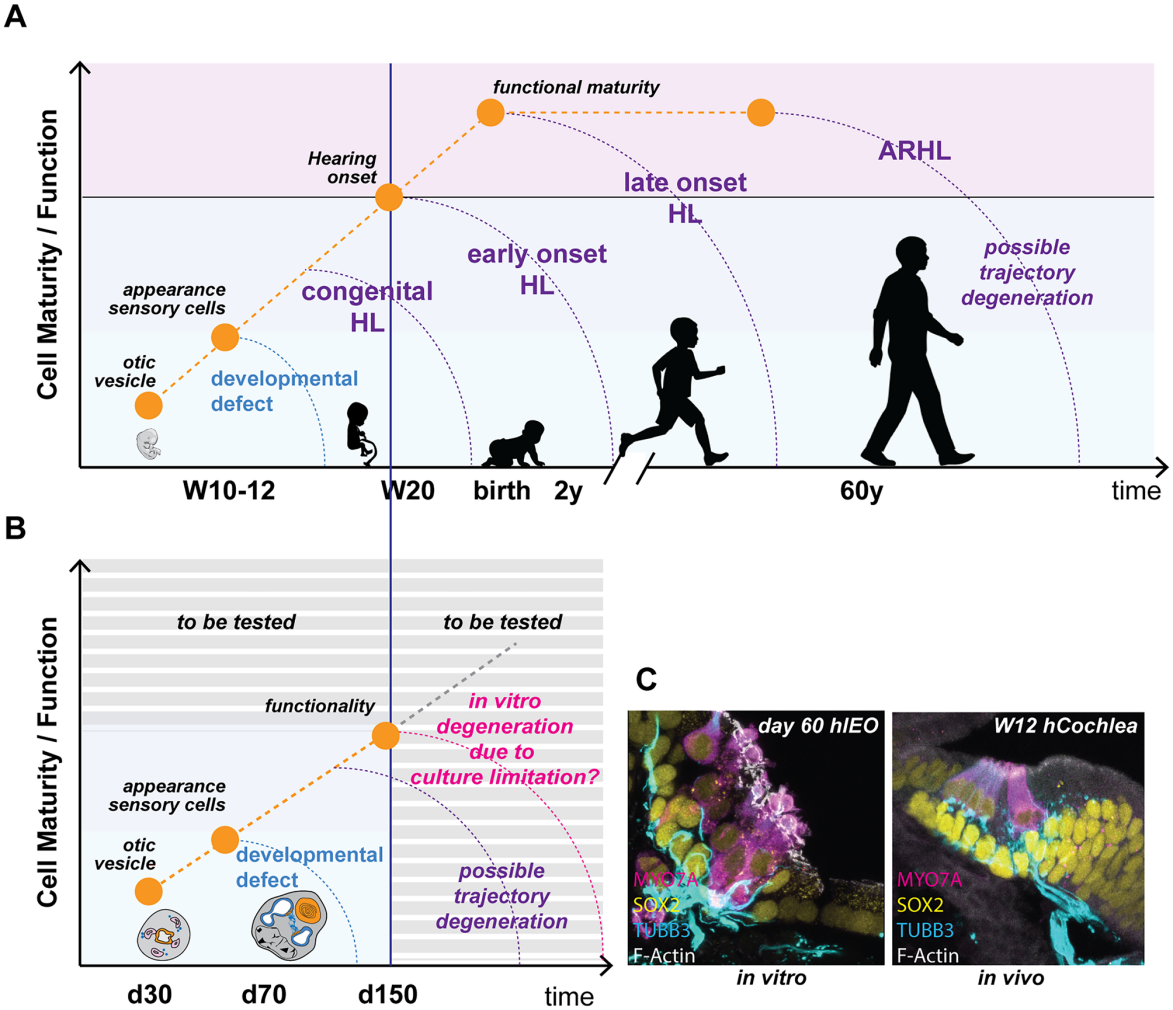
**A** Hair cells appear in vivo around W10/12 of development. Hearing onset starts during the third trimester of pregnancy. At birth, the human cochlea is mature with only a few modifications occurring subsequently [41]. Cell degeneration at different time points can result in hearing loss (HL). Early-, late-onset, and age-related hearing loss (ARHL) take place after birth. Congenital HL is caused by early degenerative events occurring still in utero. **B** The maturity level of IEOs in vitro is currently more suited to assess early defects leading to congenital HL. Potential in vitro artifacts causing culture deterioration need to be carefully evaluated and bypassed to analyze cellular phenotypes. **C** Representative example of in vitro-derived hair cells at day 60 of differentiation and cochlear hair cells at W12 of development. Adapted from Doda  et al. [14]

The fetal nature of the in vitro–derived cell types is a common feature of all human PSC-derived models [21, 22]. While the temporal window covered is somewhat limited, equivalent to the neonatal stages of mouse development, IEOs offer the unprecedented opportunity to gain insights into molecular mechanisms of human-specific organ/cell biology. Because of their “immature” stage, these models are currently more suitable to address early developmental defects leading to congenital hearing loss than late degenerative events such as age-related hearing loss (Fig. 2). Improving maturity and functionality of these cultures should be a main objective for our field.

## Modeling Developmental Defects

The corroboration that in vitro differentiation recapitulates early human organ development and that genes associated with hearing loss are expressed in IEOs [14, 16, 17] has demonstrated the possibility for the use of these models to study defects caused by gene mutations, in particular for monogenic forms of hereditary deafness. The relevant literature for this approach has recently been reviewed [3]. Notably, the differentiation potential of iPSC makes them ideal to model syndromic forms of hearing loss. By generating organoids of the different organs affected by the mutation, one could gain insight into the shared or tissue-specific molecular causes of the disease.

**Fig. 3:**
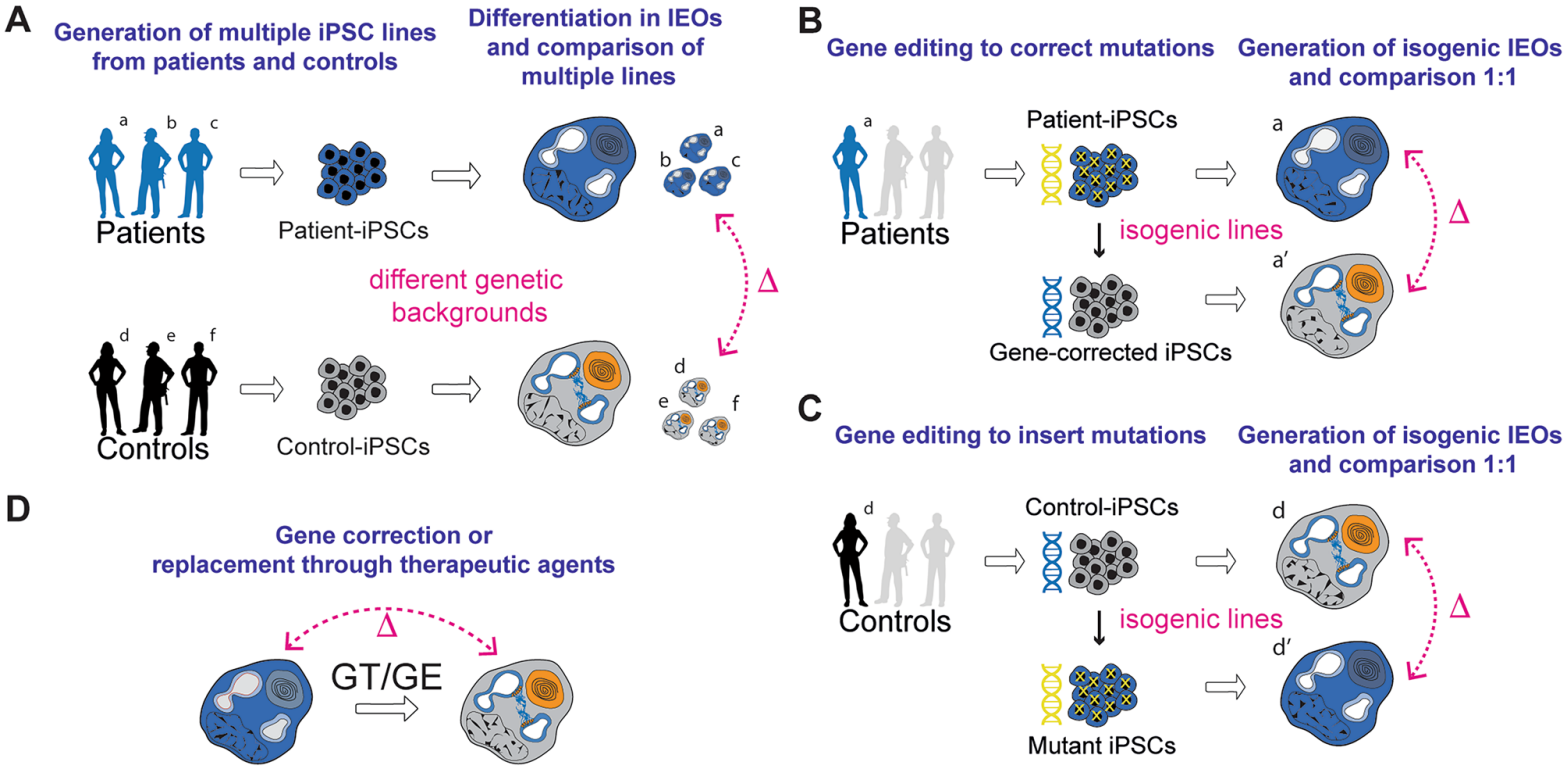
**A** The comparison between cohorts of iPSC lines (patients and controls) can be used to assess the consequences of the genetic alteration(s). Multiple lines need to be assessed as the different backgrounds are confounding factors. **B–C** Mutations can be introduced in WT lines or corrected in patient lines to generate isogenic controls. After differentiation in IEOs, phenotypes can be compared within the same genetic background. **D** Organoids generated from mutant lines can be treated with gene therapy (GT) or with gene/base editors (GE) to assess the efficacy of phenotype reversion. Pink dashed arrows and symbol Δ are used to represent different modalities to compare the generated IEOs

The absence of cell types due to developmental impairment can be difficult to demonstrate and unequivocally link to a mutation if the culture differentiation method is not optimal and suffers from variability in efficiency. The analysis of several lines and genotypes increases confidence in this respect. In addition, the use of isogenic lines as controls may reduce differences across experiments [23] (Fig. 3).

In a recent example, CRISPR/Cas9 genome editing was exploited to generate a series of mutant lines to study the molecular basis of CHARGE syndrome in IEOs [16]. Here, the authors demonstrated that otic progenitors derived in vitro from a *CHD7* knockout line fail to differentiate to sensory epithelia, potentially explaining some of the phenotypes observed in patients.

Gene mutations which result in late degenerative events are more difficult to assess in culture due to issues with maintenance of a “healthy culture” for prolonged times (Fig. 2B). For example, this was observed in the case of mutations in the *Tmprss3* gene in mESC-derived IEOs. Specifically, hair cell degeneration was detected just before the culture started to show signs of deterioration [24]. Finally, mild phenotypes such as hair bundle disorganization or cellular polarity defects may be impossible to identify in IEOs as the tissue organization does not have all the stereotypic features and patterns observed in vivo.

As our understanding and control of the system and of the culture conditions improve, addressing gross developmental defects and more subtle phenotypes will become more feasible and reliable.

## In Vitro Validation of Therapeutic Strategies for Hearing Restoration

One of the potential applications of IEOs is their use as a platform to validate therapeutic interventions on human/patient-specific cells. Several gene therapy strategies (replacement/augmentation), including gene/base editing, small molecules, and antisense oligonucleotides, have been developed in the last few decades for hearing restoration and have shown promising results in different animal models [25–28]. Testing of these approaches in IEOs could provide evidence about the transferability of findings across species and a better understanding of their safety and efficacy. Finally, IEOs could also serve as models to comply with the requirement for a reduction in animal experimentation for drug development.

IEOs contain multiple inner ear cell types, including sensory and non-sensory epithelia, otic-like neuroblasts, neurons, glia, mesenchyme, and off-target tissues co-developed in vitro, such as cartilage and epidermis. The cell composition is not generated in “fixed” relative proportions, and spatial organization lacks the consistency of the in vivo counterpart, complicating data interpretation. The type of assays one could perform should therefore be carefully evaluated to account for these current limiting factors.

State-of-the-art-IEOs are suitable for in vitro testing in cases where the cellular target is known and present in the organoid. For example, to evaluate hair cells or supporting cell transduction by adeno-associated viral (AAV) vectors and lentiviral vectors or to assess drug uptake. These types of experiments can produce a direct answer regarding cell targeting and provide insights into how to further optimize specificity. In contrast, when the cellular target is not well represented in IEOs, or is unknown, additional optimization of the model is obviously required to avoid false negative results. For example, targeting stria vascularis is likely to be unfeasible currently, as there’s little supporting evidence that a population of equivalent cells differentiates in vitro.

Finally, IEOs offer a model to test the efficacy of gene replacement/editing strategies to correct a genetic mutation. Genome editing of control iPSC lines or iPSC generation from patients’ cells can be used for the establishment of disease models and, in turn, exploited to assess therapeutic efficacy (Fig. 3D). This approach has been successfully utilized in other stem cell models [23, 29, 30], which should serve as guidance for the hearing research field.

## IEOs as a Model to Test Drug or Infection Susceptibility

In vitro toxicity screens directly on human cells allow, in principle, for the assessment of ototoxic profiles and the selection of drug candidates that lack unwanted side effects. Functional features required for the assay should however be present and robustly characterized. As of today, cells derived in vitro are fetal-like. The susceptibility of sensory cells to ototoxic drugs at this developmental stage may differ from adults. Moreover, the penetration of drugs across the whole volume of the organoids may be variable depending on the organoid composition and depth of the sensory vesicles. Once these limitations are overcome, IEOs could be used for in vitro testing.

Besides drug ototoxicity, one could also use organoids to assess the effect of infections of pathogenic viruses such as cytomegalovirus (CMV), bypassing the difficulty in accessing postmortem tissues [31], with the advantage of controlled experimental conditions. A recent example analyzed Sars-Cov-2 infection in IEOs [32].

Further optimization is certainly needed to upscale IEOs and in general iPSC-based organoids to medium or high-throughput screening [33, 34]. A first example of a large drug screen (2700 compounds in 20,000 organoids) in human iPSC-derived retinal organoids has been recently reported tackling cone photoreceptor survival. Retinal organoids are among the first models that have been developed [35], are now very advanced, and have been extensively characterized [36]. These studies demonstrate the feasibility of such approaches and provide a roadmap for application in other organoid models.

## Assay Readouts

iPSC-derived organoids are usually complex in term of cell composition and large in size and assays based on them require critical thinking [37].

Histological analysis of organoids at selected endpoints is very laborious and low throughput. While sufficient for qualitative assessment, quantification biases can be introduced if not all sections are considered. Alternatively, one could use reporter lines to monitor the presence and quantify the number of selected cell types [12, 38]. Live-fluorescence may be difficult to evaluate with image-based methods in large organoids due to light scattering but could facilitate quantification by alternative approaches, such as flow cytometry.

Single-cell or single nuclei RNA-sequencing approaches are particularly suited to address the issue of organoid heterogeneity, as they enable a refined analysis of the relative abundance of cell types and if/how their gene expression is affected by a genetic mutation or drug treatment [23]. However, cell isolation methods to generate single-cell suspension from organoids could favor the purification of loosely adherent populations at the expenses of tight epithelia, or be too harsh for some cells, resulting in their under-representation.

Finally, when the focus of the experiments goes beyond presence/absence (death/survival) of a selected cell type and the treatment may impact cellular activity/function, ad hoc readouts to measure such effects would need to be implemented. Classical methods for hair cell electrophysiology have been used successfully in IEOs [11, 12], but are difficult to translate to high- or medium-throughput assays. Functional readouts for neuronal activity on two- and three-dimensional multielectrode arrays have been used for brain organoids [39]. These methods may not translate well to IEO cultures where neurons are only a fraction of all cells. Alternatively, genetically encoded calcium indicators such as GCaMP [40] or other biosensors could be used to monitor cellular activity. A careful evaluation of the different quantification methods and their robustness should be performed in order to set up meaningful assays.

## Conclusion

**Fig. 4:**
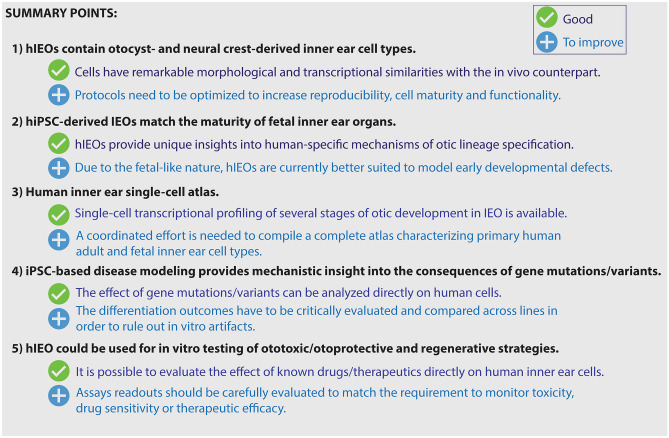
Summary points discussed in this article including IEO “features” that are already available (green) and that still need to be implemented (blue)

IEOs represent complementary tools to animal models to tackle human-specific features of inner ear development and disease (Fig. 4). While at present, IEOs can be used to validate the effect of known mutations and therapeutics on human cells, in the future, they may enable discoveries. This will require more robust protocols, reference atlases for the developing inner ear, optimization of the readouts, and the integration of cutting-edge techniques like co-cultures on organ-on-chip. The establishment of international consortia, the sharing of patient-derived, engineered, reporter lines, and protocols should be reinforced, also by funding agencies, to facilitate the uptake of this technology by different laboratories and to enable this promising field to progress at a rapid pace.
